# Hospital Outcomes among COVID-19 Hospitalizations with Acute Ischemic Stroke: Cross-Sectional Study Results from California State Inpatient Database

**DOI:** 10.3390/brainsci12091177

**Published:** 2022-09-01

**Authors:** Muni Rubens, Anshul Saxena, Venkataraghavan Ramamoorthy, Md Ashfaq Ahmed, Zhenwei Zhang, Peter McGranaghan, Emir Veledar, Michael McDermott, Felipe De Los Rios La Rosa

**Affiliations:** 1 Miami Cancer Institute, Baptist Health South Florida, Miami, FL 33176, USA; 2 Center for Advanced Analytics, Baptist Health South Florida, Miami, FL 33176, USA; 3 Department of Neurology, Florida International University, Miami, FL 33199, USA; 4 Department of Internal Medicine and Cardiology, Charité—Universitätsmedizin Berlin, Corporate Member of Freie Universität Berlin and Humboldt Universität zu Berlin, 10117 Berlin, Germany; 5 Miami Neuroscience Institute, Baptist Health South Florida, Miami, FL 33176, USA

**Keywords:** coronavirus, death, hospitalization, ischemic stroke, morbidity

## Abstract

Coronavirus disease 2019 (COVID-19) could be a risk factor for acute ischemic stroke (AIS) due to the altered coagulation process and hyperinflammation. This study examined the risk factors, clinical profile, and hospital outcomes of COVID-19 hospitalizations with AIS. This study was a retrospective analysis of data from California State Inpatient Database (SID) during 2019 and 2020. COVID-19 hospitalizations with age ≥ 18 years during 2020 and a historical cohort without COVID-19 from 2019 were included in the analysis. The primary outcomes studied were in-hospital mortality and discharge to destinations other than home. There were 91,420 COVID-19 hospitalizations, of which, 1027 (1.1%) had AIS. The historical control cohort included 58,083 AIS hospitalizations without COVID-19. Conditional logistic regression analysis showed that the odds of in-hospital mortality, discharge to destinations other than home, DVT, pulmonary embolism, septic shock, and mechanical ventilation were significantly higher among COVID-19 hospitalizations with AIS, compared to those without AIS. The odds of in-hospital mortality, DVT, pulmonary embolism, septic shock, mechanical ventilation, and respiratory failure were significantly higher among COVID-19 hospitalizations with AIS, compared to AIS hospitalizations without COVID-19. Although the prevalence of AIS was low among COVID-19 hospitalizations, it was associated with higher mortality and greater rates of discharges to destinations other than home.

## 1. Introduction

Coronavirus disease 2019 (COVID-19) has affected nearly 490 million people and resulted in more than 6 million deaths globally [1]. Due to variable presentation of the disease, understanding the effects of COVID-19 on general survival as well as organ systems has been challenging [2].

Though COVID-19 significantly increases the risk for acute ischemic stroke (AIS), similar to other viral systemic respiratory infections, studies examining these associations are relatively few [3,4]. A single center, retrospective study of 219 COVID-19 hospitalizations reported that 4.9% of these patients had AIS [5]. Similarly, many studies have reported simultaneous occurrences of severe respiratory involvement and cerebrovascular events among COVID-19 hospitalizations [6,7,8]. These associations have been hypothesized to be due to the prothrombotic effects of the hyperinflammatory response and cytokine storm observed in COVID-19. The heightened risk for AIS in COVID-19 patients is multifactorial and associated with several altered prothrombotic mechanisms such as stimulation of the coagulation process, as indicated by higher levels of fibrin and D-dimer, and hyperinflammatory states, indicated by elevated levels of lactate dehydrogenase and erythrocyte sedimentation rate, and circulatory WBC depletion [7,9].

Experts in stroke and cerebrovascular disorders have recommended that future studies should identify risk factors, clinical presentation, management strategies, and other outcomes among COVID-19 patients with AIS to better understand AIS among COVID-19 patients. Nevertheless, evidence to support clinical and practical implications are scarce. The main objective of this study was to understand the risk factors, clinical profile, and hospital outcomes of COVID-19 hospitalizations with AIS compared to those without AIS using the California State Inpatient Database (SID). In addition, we also compared the hospital outcomes of AIS patients with and without COVID-19. We selected the California SID because this state ranks first within the US in terms of the number of recorded COVID-19 cases as well as deaths [1].

## 2. Methods

### 2.1. Study Design and Data Source

This study was exempted from Institutional Review Board approval and waived the requirement for informed consent because it uses previously collected de-identified data stored in the SID.

We conducted a retrospective analysis of data from the 2019 and 2020 California SIDs. The Agency for Healthcare Research and Quality (AHRQ) developed the SIDs, which gather statewide inpatient clinical data from patients admitted to participating hospitals within the states [10]. The SID annually collects discharge data from more than 90% of patients admitted to community hospitals in participating states. The SIDs in every state include both clinical and nonclinical variables which are extracted from discharge billing data and consists of information from patients with all types of coverages such as Medicare, Medicaid, private insurance, and those who are uninsured. All clinical diagnoses were done by physicians in the participating hospitals. The SID is relatively clean and only requires a small amount of cleaning but extensive recoding based on the study requirements. We used the Strengthening the Reporting of Observational Studies in Epidemiology (STROBE) guideline for improving the quality of this study [11].

### 2.2. Study Population

All adult patients ≥ 18 years of age with COVID-19 hospitalizations during 2020 were included in the analysis. These COVID-19 hospitalizations were then grouped into those with and those without AIS. The hospitalizations were selected based on the primary diagnosis of COVID-19. Subsequently, among these hospitalizations, those with AIS were identified. We also included a historical cohort of AIS patients without COVID-19 from 2019 SID data. Hospitalizations and procedures were identified using the International Classification of Diseases, Tenth Revision, Clinical Modification (ICD-10-CM) diagnosis and procedure codes. ICD-10-CM diagnosis code U071 was used to identify COVID-19 hospitalizations, while I63, I65, and I66 were used for AIS.

### 2.3. Study Variables and Outcomes

The primary outcomes studied were in-hospital mortality and discharge to destinations other than home. These destinations included transfer to home health care, short-term hospital skilled nursing facility, intermediate care facility, or other types of facilities. The secondary outcomes were deep venous thrombosis (DVT), pulmonary embolism, systemic inflammatory response syndrome, septic shock, mechanical ventilation, and respiratory failure. Demographic variables included age, sex, race, and insurance status, while clinical risk profile included comorbidities such as hypertension, diabetes mellitus, hyperlipidemia, obesity, atrial fibrillation, coagulation disorder, peripheral vascular disease, liver disease, chronic renal failure, tobacco use, alcohol abuse, drug abuse, congestive heart failure (CHF), prior myocardial infarction (MI), prior percutaneous coronary intervention (PCI), and prior coronary artery bypass graft surgery (CABG), and Elixhauser comorbidity index. We used ICD-10-CM diagnosis and procedure codes to identify these variables (Supplementary Table S1).

Since we used a database that was already de-identified and publicly available, Baptist Health South Florida’s institutional review board approved this study and waived the need for informed consent. We followed the Strengthening the Reporting of Observational Studies in Epidemiology (STROBE) reporting guideline for this study.

### 2.4. Statistical Analysis

Data were extracted by trained biostatisticians and converted to SAS format and merged before recoding and analyses. Descriptive statistics were used to identify the differences in demographics and clinical profiles between COVID-19 hospitalizations with and without AIS and AIS hospitalizations without COVID-19. Continuous variables were reported as medians and interquartile ranges, while categorical variables were reported as frequencies and percentages. The *t*-test and Mann–Whitney U test were used to compare continuous variables, while the Rao–Scott chi-square test was used to compare categorical variables.

We controlled for possible differences in demographic and clinical profiles between the three groups using the propensity score matching (PSM) process. We conducted two separate PSMs, one for comparison between COVID-19 hospitalizations with AIS versus COVID-19 hospitalizations without AIS, and another for COVID-19 hospitalizations with AIS versus AIS hospitalizations without COVID-19. PSM included nonparsimonious multivariable logistic regression, which accounts for nonrandom treatment selection using propensity scores estimated for all hospitalizations based on demographic characteristics and clinical profiles. Sex, hypertension, diabetes mellitus, hyperlipidemia, obesity, atrial fibrillation, coagulation disorder, peripheral vascular disease, liver disease, chronic renal failure, tobacco use, alcohol abuse, drug abuse, CHF, prior MI, prior PCI, and prior CABG were included in the model. A 1:1 greedy matching algorithm with a caliper of 0.25 times the standard deviation of the logit of the propensity score was used for matching. The standardized mean differences in the distribution of covariates were set at <10% to assure sufficient matching.

Multivariate conditional logistic regression analyses with enter method were used to estimate the odds of in-hospital mortality, discharge to destinations other than home, DVT, pulmonary embolism, systemic inflammatory response syndrome, septic shock, mechanical ventilation, and respiratory failure between COVID-19 hospitalizations with AIS and COVID-19 hospitalizations without AIS. These regression models were adjusted for covariates such as age, sex, race, insurance, hypertension, diabetes mellitus, hyperlipidemia, obesity, atrial fibrillation, coagulation disorder, peripheral vascular disease, liver disease, chronic renal failure, tobacco use, alcohol abuse, drug abuse, CHF, prior MI, prior PCI, and prior CABG. Similarly, we conducted multivariate conditional logistic regression analyses for estimating the odds of the same outcomes between COVID-19 hospitalizations with AIS versus AIS hospitalizations without COVID-19, controlling for the same covariates. Missing data were <5% and were excluded from the analysis. Statistical significance was set at *p* < 0.05 and all tests were two sided. All statistical analyses were conducted using SAS software, version 9.4 (SAS Inc., Cary, NC, USA).

## 3. Results

We included a total of 91,420 COVID-19 hospitalizations for the analysis, of which, 1027 (1.1%) had AIS. In addition, we also included 58,083 AIS hospitalizations without a diagnosis of COVID-19 from 2019 data. Among COVID-19 hospitalizations with AIS, the majority were in the age group ≥ 65 years (70.2%) and men (60.0%). Hispanics constituted the majority of these hospitalizations (42.0%), followed by Whites (35.6%), Asians, Pacific Islanders, Native Americans (9.8%), and Blacks (7.6%). The majority had Medicare (66.7%), followed by Medicaid (17.1%) and private insurance (12.9%) coverage, while 1.4% were uninsured. The most common comorbidities were hypertension (82.1%), hyperlipidemia (44.2%), chronic renal failure (31.7%), congestive heart failure (24.9%), and coagulation disorders (23.4%). The majority of these hospitalizations (85.9%) had an Elixhauser comorbidity index ≥ 3. There were significant differences in all demographic characteristics between COVID-19 hospitalizations with and without AIS (Table 1). The prevalence of comorbidities such as hypertension, atrial fibrillation, coagulation disorder, peripheral vascular disease, chronic renal failure, congestive heart failure, prior MI, prior PCI, and prior CABG were significantly higher among COVID-19 hospitalizations with AIS, while diabetes mellitus, hyperlipidemia, and obesity were significantly higher among COVID-19 hospitalizations without AIS. A majority of the COVID-19 hospitalizations with AIS (96.1%) did not receive AIS treatments such as intravenous thrombolysis or mechanical thrombectomy.

Demographic characteristics between COVID-19 hospitalizations with AIS and AIS hospitalizations without COVID-19 showed significant differences only in sex and race (Table 1). The proportion of males among COVID-19 hospitalizations with AIS was significantly greater than AIS hospitalizations without COVID-19 (60.0% versus 53.3%, *p* < 0.001). Among COVID-19 hospitalizations with AIS, a majority of the hospitalizations were among Hispanics (42.0%), followed by Whites (35.6%), Asians, Pacific Islanders, Native Americans (9.8%), and Blacks (7.6%). Among AIS hospitalizations without COVID-19, a majority of the hospitalizations were among Whites (50.4%), followed by Hispanics (23.7%), Asians, Pacific Islanders, Native Americans (11.9%), and Blacks (9.5%). A comparison of the clinical risk profiles showed that obesity, coagulation disorder, peripheral vascular disease, liver disease, chronic renal failure, and congestive heart failure were significantly higher among COVID-19 hospitalizations with AIS while, hypertension, hyperlipidemia, tobacco use, alcohol abuse, and drug abuse were significantly higher among AIS hospitalizations without COVID-19. A majority of the AIS hospitalizations without COVID-19 (81.1%) did not receive intravenous thrombolysis or mechanical thrombectomy.

**Figure 1:**
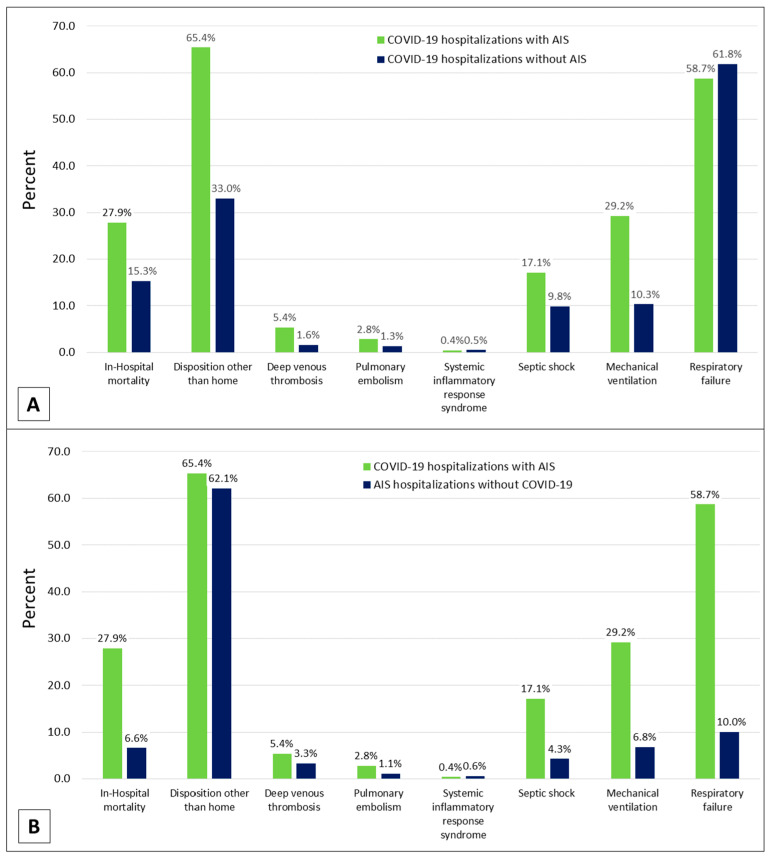
Comparison of adverse hospital outcomes between (**A**) COVID-19 hospitalizations with AIS and COVID-19 hospitalizations without AIS and between (**B**) COVID-19 hospitalizations with AIS and AIS hospitalizations without COVID-19.

Hospital outcomes such as in-hospital mortality (27.9% versus 15.3, *p* < 0.001), discharge to destinations other than home (65.4% versus 33.0%, *p* < 0.001), DVT (5.4% versus 1.6%, *p* < 0.001), pulmonary embolism (2.8% versus 1.3%, *p* = 0.012), septic shock (17.1% versus 9.8%, *p* < 0.001), and mechanical ventilation (29.2% versus 10.3%, *p* < 0.001), were significantly higher among COVID-19 hospitalizations with AIS, compared to COVID-19 hospitalizations without AIS (Figure 1A).

Likewise, in-hospital mortality (27.9% versus 6.6%, *p* < 0.001), DVT (5.4% versus 3.3%, *p* = 0.022), pulmonary embolism (2.8% versus 1.1%, *p* = 0.004), septic shock (17.1% versus 4.3%, *p* < 0.001), mechanical ventilation (29.2% versus 6.8%, *p* < 0.001), and respiratory failure (58.7% versus 10.0%, *p* < 0.001), were significantly higher among COVID-19 hospitalizations with AIS, compared to AIS hospitalizations without COVID-19 (Figure 1B).

**Figure 2:**
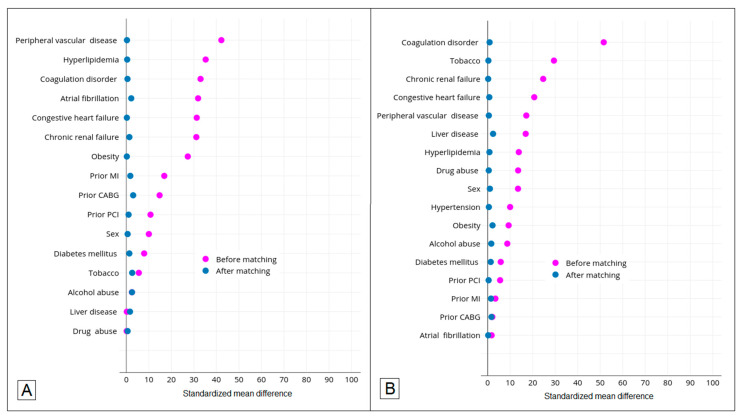
Standardized mean differences between variables before and after propensity score matching for (**A**) COVID-19 hospitalizations with AIS and COVID-19 hospitalizations without AIS and for (**B**) COVID-19 hospitalizations with AIS and AIS hospitalizations without COVID-19.

Conditional logistic regression analysis showed that the odds of in-hospital mortality (OR, 2.16; 95% CI, 1.72–2.73), discharge to destinations other than home (OR, 2.61; 95% CI, 2.10–3.25), DVT (OR, 3.43; 95% CI, 2.03–5.80), pulmonary embolism (OR, 2.30; 95% CI, 1.27–4.16), septic shock (OR, 2.07; 95% CI, 1.57–2.74), and mechanical ventilation (OR, 4.69; 95% CI, 3.56–6.16) were significantly higher among COVID-19 hospitalizations with AIS, compared to COVID-19 hospitalizations without AIS (Table 2). Likewise, the odds of in-hospital mortality (OR, 5.65; 95% CI, 4.20–7.61), DVT (OR, 1.82; 95% CI, 1.14–2.91), pulmonary embolism (OR, 2.61; 95% CI, 1.24–5.49), septic shock (OR, 4.74; 95% CI, 3.29–6.84), mechanical ventilation (OR, 6.17; 95% CI, 4.55–8.38), and respiratory failure (OR, 15.37; 95% CI, 11.86–19.92) were significantly higher among COVID-19 hospitalizations with AIS, compared to AIS hospitalizations without COVID-19 (Table 3). Propensity score matching for the analysis successfully yielded covariate balance as evidenced by a standardized mean difference of <10% for all covariates after matching (Figure 2).

## 4. Discussion

We used the California SID to search for the effects of AIS on adverse hospital outcomes among COVID-19 hospitalizations. California ranks the highest within the US in terms of the total number of COVID-19 cases as well as COVID-19 deaths. To the best of our knowledge, this is one of the few large-scale studies that searched for these effects using a large and representative database. We found that adverse hospital outcomes such as in-hospital mortality and discharge to destinations other than home were significantly higher among COVID-19 hospitalizations with AIS and could be due to COVID-19 itself or pre-existing cardiovascular risk factors.

The prevalence of AIS among COVID-19 hospitalizations in our study was 1.1%. Previous studies have reported varying prevalences ranging from 0.9% to 5%. For example, in a study of data from 54 health care facilities, the prevalence of AIS among COVID-19 patients was 1.3% [12]. In a study during the initial spread of the pandemic, the incidence of acute cerebrovascular diseases was as high as 5% among COVID-19 patients with severe forms of the disease [7]. In a retrospective cohort study that measured the incidence of AIS after COVID-19 infection, 0.9% had imaging proven ischemic stroke [13]. Given these varying prevalences, the findings in our study are significant because we used a substantially large sample size of 91,420 COVID-19 hospitalizations for calculating our estimates. Therefore, our estimates could be considered to be more accurate and generalizable.

We found that most of the COVID-19 hospitalizations with AIS had greater levels of cardiovascular risk factors such as hypertension, atrial fibrillation, coagulation disorders, peripheral vascular disease, chronic renal failure, congestive heart failure, tobacco use, and prior coronary artery disease. These findings were also similar in AIS hospitalizations without COVID-19. However, it is worth noting that COVID-19 patients in general had greater levels of coagulation disorders, compared to AIS hospitalizations without COVID-19. COVID-19 patients with history of coagulation disorders are at increased risk of severe forms of the disease and mortality [14]. In addition, COVID-19 itself is a risk factors for venous thromboembolism and arterial thrombosis, also called as COVID-19-associated coagulopathy (CAC) [15]. CAC is riskier than disseminated intravascular coagulation (DIC) and sepsis-induced coagulopathy (SIC) as although it initially presents with insignificant changes in prothrombin time and platelet count, it rapidly progresses to significantly higher levels of D-dimer and fibrinogen, and increases the risk for AIS [15]. The findings in our study contradict some of the earlier studies which showed that AIS among COVID-19 hospitalizations occurred among younger patients without significant cardiovascular risk factors [16,17]. Alternatively, recent large-scale studies have also shown that though COVID-19 could be considered an independent risk factor, AIS occurs predominantly among patients with preexisting cardiovascular risk factors [9,18].

We found that adverse hospital outcomes such as in-hospital mortality and adverse discharge dispositions were significantly higher among COVID-19 hospitalizations with AIS, compared to those without AIS. These findings are also found in other studies. A retrospective cohort study showed that AIS was significantly associated with greater in-hospital mortality among COVID-19 hospitalizations [12]. This study also showed that the risk of discharge to a destination other than home doubled with simultaneous occurrence of AIS and COVID-19. Similarly, in a study among COVID-19 patients ≤50 years of age, survival analysis showed that stroke patients had significantly lower odds of survival, compared to those without stroke [19]. Greater rates of mortality and adverse discharge dispositions in this population could be due to multisystemic causes such respiratory involvement, circulatory collapse, and coagulation disorders. We also found that in-hospital mortality and adverse discharge dispositions were significantly higher among COVID-19 hospitalizations with AIS, compared to AIS hospitalizations without COVID-19. These findings suggests that COVID-19 itself could be a contributing factor for higher mortality and adverse discharge dispositions among those with AIS. Similar to our findings, a retrospective cohort study showed that the survival probability was significantly lower among ischemic stroke patients with COVID-19, compared to historical AIS controls without COVID-19 [20]. A retrospective study by Qureshi et al. found that the rates of discharge to destinations other than home were higher among patients with COVID-19 and AIS, compared to AIS alone [12]. This could be explained by the fact that COVID-19 causes elevated inflammation and an altered coagulation profile, which itself increases the morbidity and mortality risk among AIS patients [6,9]. In addition, among AIS patients with COVID-19, the intensity of neuronal injury is greater and the responses to existing treatments are poor [16,21].

AIS among COVID-19 patients frequently goes unnoticed due to the rarity of the condition and variable presentation of COVID-19. In addition, AIS is also seen among COVID-19 patients without severe forms of the disease [4]. The coexistence of these two conditions impairs the chances of recovery, survival, and safe discharge to home. Given these adverse associations and the likelihood to go undetected, all COVID-19 patients should be suspected of signs of AIS for early identification and management. The greater rates of mortality and adverse dispositions among COVID-19 patients with AIS could be due to multisystem failure. It is also less likely that treatments for AIS could have increased these adverse outcomes, given the relatively small number of COVID-19 patients receiving AIS treatments in our study. Assessing the levels of multisystem failure early during COVID-19 could help in risk stratifying COVID-19 patients who are at greater risk of experiencing adverse outcomes due to AIS. This would also help in aggressive management of COVID-19-related adverse outcomes in AIS. In addition, delays in AIS detection and treatment due to changes in existing triage guidelines and the time lag between diagnosis and treatment due to COVID-19 containment measures should be overcome by prioritizing resources for these high-risk patients [22,23].

## 5. Limitations

Our study has some limitations. We used the California SID for our analysis. The SID, being an administrative database, does not have information on the severity of AIS or COVID-19, diagnostic and prognostic tests, laboratory findings, medications, or imaging results. Therefore, we could not ascertain the reasons for differences in outcomes across different groups within our study. Availability of such information could have significantly improved the findings in our study. We used ICD-10 diagnosis and procedure codes for identifying conditions and procedures, which could have resulted in some coding errors leading to misclassification bias. We used COVID-19 data collected during 2020, and there have been significant advancements in the understanding and management of the disease since then. Therefore, the findings in our study should be cautiously interpreted. Since the SID contains data from hospitalized patients only, our findings are not generalizable to non-hospitalized patients with conditions such as transient ischemic attack and non-severe forms of ischemic strokes. The lack of data on non-hospitalized patients is significantly limiting because a majority of non-severe ischemic stroke patients would have avoided hospitalization to prevent COVID-19 transmission.

## 6. Conclusions

We found that adverse hospital outcomes such as in-hospital mortality, discharge to destinations other than home, deep venous thrombosis, pulmonary embolism, systemic inflammatory response syndrome, septic shock, mechanical ventilation, and respiratory failure were significantly higher among COVID-19 hospitalizations with AIS, compared to those without AIS. Similarly, in-hospital mortality, DVT, pulmonary embolism, septic shock, mechanical ventilation, and respiratory failure were significantly higher among COVID-19 hospitalizations with AIS, compared to AIS hospitalizations without COVID-19. Our study confirms the findings of previous studies that though the prevalence of AIS among COVID-19 hospitalizations is low, it is associated with adverse hospital outcomes. These findings could be due to COVID-19 itself or pre-existing cardiovascular risk factors. Since AIS among COVID-19 patients is infrequent but associated with adverse hospital outcomes, COVID-19 patients with AIS should be aggressively managed to prevent complications. Future studies should explore the mechanisms for the adverse associations found in our study.

## Figures and Tables

**Table 1:** Demographic and clinical characteristics of hospitalized COVID-19 patients with and without AIS.

Characteristic	COVID-19 Hospitalizations with AIS n = 1027 (1.1%)	COVID-19 Hospitalizations without AIS n = 90,393 (98.9%)	*p* Value ^a^	AIS Hospitalizations without COVID-19 n = 58,083 (98.9%)	*p* Value ^b^
Age, n (%)			<0.001		0.270
18–44 years	44 (4.3%)	14,923 (16.5%)		2346 (4.0%)	
45–64 years	262 (25.5%)	35,121 (38.9%)		16,132 (27.8%)	
≥65 years	721 (70.2%)	40,349 (44.6%)		39,605 (68.2%)	
Sex, n (%)			0.001		<0.001
Male	616 (60.0%)	49,745 (55.0%)		30,966 (53.3%)	
Female	411 (40.0%)	40,645 (45.0%)		27,114 (46.7%)	
Race, n (%)			<0.001		<0.001
White	360 (35.6%)	22,847 (25.7%)		28,909 (50.4%)	
Black	77 (7.6%)	5114 (5.7%)		5456 (9.5%)	
Hispanic	424 (42.0%)	48,250 (54.2%)		13,579 (23.7%)	
Asian, Pacific Islander, Native American	99 (9.8%)	8274 (9.3%)		6855 (11.9%)	
Other	50 (5.0%)	4535 (5.1%)		2587 (4.5%)	
Insurance, n (%)			<0.001		0.050
Medicare	685 (66.7%)	37,966 (42.0%)		37,047 (63.8%)	
Medicaid	176 (17.1%)	25,713 (28.5%)		9497 (16.4%)	
Private insurance	132 (12.9%)	21,833 (24.2%)		9297 (16.0%)	
Uninsured	14 (1.4%)	1668 (1.8%)		1095 (1.9%)	
Other	20 (1.9%)	3169 (3.5%)		1134 (2.0%)	
Clinical risk profile, n (%)					
Hypertension	843 (82.1%)	54,659 (60.5%)	<0.001	49,803 (85.7%)	<0.001
Diabetes mellitus	144 (14.0%)	15,264 (16.9%)	0.014	9350 (16.1%)	0.072
Hyperlipidemia	454 (44.2%)	55,623 (61.5%)	<0.001	36,346 (62.6%)	<0.001
Obesity	176 (17.1%)	25,751 (28.5%)	<0.001	8000 (13.8%)	0.002
Atrial fibrillation	214 (20.8%)	8622 (9.5%)	<0.001	11,690 (20.1%)	0.573
Coagulation disorder	240 (23.4%)	10,018 (11.1%)	<0.001	3343 (5.8%)	<0.001
Peripheral vascular disease	207 (20.2%)	5595 (6.2%)	<0.001	7980 (13.7%)	<0.001
Liver disease	62 (6.0%)	5409 (6.0%)	0.943	1517 (2.6%)	<0.001
Chronic renal failure	326 (31.7%)	16,655 (18.4%)	<0.001	12,174 (21.0%)	<0.001
Tobacco use	60 (5.8%)	4162 (4.6%)	0.060	8528 (14.7%)	<0.001
Alcohol abuse	27 (2.6%)	2030 (2.2%)	0.410	2447 (4.2%)	0.012
Drug abuse	30 (2.9%)	2615 (2.9%)	0.957	3285 (5.7%)	<0.001
Congestive heart failure	256 (24.9%)	11,601 (12.8%)	<0.001	9629 (16.6%)	<0.001
Prior MI	71 (6.9%)	2917 (3.2%)	<0.001	4537 (7.8%)	0.287
Prior PCI	50 (4.9%)	2536 (2.8%)	<0.001	3559 (6.1%)	0.094
Prior CABG	54 (5.3%)	2192 (2.4%)	<0.001	3328 (5.7%)	0.518
Elixhauser comorbidity index, n (%)			---		---
0	---	6712 (7.4%)		1668 (2.9%)	
1 or 2	140 (13.6%)	31,371 (34.7%)		21,444 (36.9%)	
≥3	882 (85.9%)	52,310 (57.9%)		34,971 (60.2%)	
Treatment			---		---
Intravenous thrombolysis only	0 (0%)	---		0 (0%)	
Mechanical thrombectomy only	---	---		4289 (7.4%)	
Both	32 (3.1%)	---		6681 (11.5%)	
None	987 (96.1%)	---		47,113 (81.1%)	

Abbreviations: AIS, acute ischemic stroke; MI, myocardial infarction; PCI, percutaneous coronary intervention; CABG, coronary artery bypass grafting. ^a^ *p* value corresponds to comparison between COVID-19 hospitalizations with AIS and COVID-19 hospitalizations without AIS. ^b^ *p* value corresponds to comparison between COVID-19 hospitalizations with AIS and AIS hospitalizations without COVID-19. Missing data is because cell sizes was less than or equal to 10 to comply with the requirements of Healthcare Cost and Utilization Project (HCUP).

**Table 2:** Conditional logistic regression of hospital outcomes between COVID-19 hospitalizations with AIS and COVID-19 hospitalizations without AIS.

Characteristic	Odds Ratio	*p* Value
In-Hospital mortality	2.16 (1.72–2.73)	<0.001
Disposition other than home	2.61 (2.10–3.25)	<0.001
Deep venous thrombosis	3.43 (2.03–5.80)	<0.001
Pulmonary embolism	2.30 (1.27–4.16)	0.006
Systemic inflammatory response syndrome	0.93 (0.38–2.30)	0.877
Septic shock	2.07 (1.57–2.74)	<0.001
Mechanical ventilation	4.69 (3.56–6.16)	<0.001
Respiratory failure	0.86 (0.72–1.03)	0.102

Odds ratios were calculated using conditional logistic regression after adjusting for age, sex, race, insurance, hypertension, diabetes mellitus, hyperlipidemia, obesity, atrial fibrillation, coagulation disorder, peripheral vascular disease, liver disease, chronic renal failure, tobacco use, alcohol abuse, drug abuse, congestive heart failure, prior MI, prior PCI, and prior CABG. Odds ratio corresponds to COVID-19 hospitalizations with AIS, with COVID-19 hospitalizations without AIS as the reference. Complete model results are available from the authors upon request.

**Table 3:** Conditional logistic regression of hospital outcomes between COVID-19 hospitalizations with AIS and AIS hospitalizations without COVID-19.

Characteristic	Odds Ratio	*p* Value
In-Hospital mortality	5.65 (4.20–7.61)	<0.001
Disposition other than home	1.19 (0.96–1.48)	0.110
Deep venous thrombosis	1.82 (1.14–2.91)	0.012
Pulmonary embolism	2.61 (1.24–5.49)	0.011
Systemic inflammatory response syndrome	0.73 (0.30–1.79)	0.490
Septic shock	4.74 (3.29–6.84)	<0.001
Mechanical ventilation	6.17 (4.55–8.38)	<0.001
Respiratory failure	15.37 (11.86–19.92)	<0.001

Odds ratios were calculated using conditional logistic regression after adjusting for age, sex, race, insurance, hypertension, diabetes mellitus, hyperlipidemia, obesity, atrial fibrillation, coagulation disorder, peripheral vascular disease, liver disease, chronic renal failure, tobacco use, alcohol abuse, drug abuse, congestive heart failure, prior MI, prior PCI, and prior CABG. Odds ratio corresponds to COVID-19 hospitalizations with AIS, with AIS hospitalizations without COVID-19 as the reference. Complete model results are available from the authors upon request.

## Data Availability

Data is publicly available for purchase at: https://www.hcup-us.ahrq.gov/sidoverview.jsp (accessed on 24 August 2022).

## Supplementary Materials

The following supporting information can be downloaded at: https://www.mdpi.com/article/10.3390/brainsci12091177/s1, Table S1. ICD-10 codes.
