# Supplementary material for: Hospital Outcomes among COVID-19 Hospitalizations with Acute Ischemic Stroke: Cross-Sectional Study Results from California State Inpatient Database
Source: Brain Sci. 2022 Sep 1;12(9):1177. doi: 10.3390/brainsci12091177 (PMC9496747; doi:10.3390/brainsci12091177)
Supplement: Supplementary file 1 [file brainsci-12-01177-s001.zip › brainsci-1803119-supplementary.pdf]

Table S1. ICD-10 codes.

Variables	ICD-10 codes
AIS	I63, I65, and I66
Cerebral edema	G935 and G93.6
Deep venous thrombosis	I82
Pulmonary embolism	I26
Intracerebral hemorrhage	I61 and I629
Systemic inflammatory response syndrome	R651
Septic shock	A41 and R6521
Mechanical ventilation	5A1945Z, 5A1955Z, 0BH17EZ, and 0BH18EZ
Respiratory failure	J96
